# The Changes of Amygdala Transcriptome in Autism Rat Model After Arginine Vasopressin Treatment

**DOI:** 10.3389/fnins.2022.838942

**Published:** 2022-03-25

**Authors:** Bo Zhou, Xiaoli Zheng, Yunhua Chen, Xuehui Yan, Jinggang Peng, Yibu Liu, Yi Zhang, Lei Tang, Min Wen

**Affiliations:** ^1^State Key Laboratory of Functions and Applications of Medicinal Plants, Guizhou Medical University, Guiyang, China; ^2^Guizhou Provincial Engineering Technology Research Center for Chemical Drug R&D, Guizhou Medical University, Guiyang, China; ^3^College of Pharmacy, Guizhou Medical University, Guiyang, China; ^4^College of Basic Medical, Guizhou Medical University, Guiyang, China

**Keywords:** autism spectrum disorder, neurodevelopmental, arginine vasopressin, amygdala, oligodendrocyte

## Abstract

**Background:**

Some studies have shown that arginine vasopressin (AVP) can significantly improve the social interaction disorder of autism, but the mechanism remains unclear.

**Methods:**

Female Wistar rats were intraperitoneally injected with VPA or normal saline at embryonic day 12.5 to establish an autism model or normal control in their offspring. Male offspring prenatally exposed to VPA were randomly assigned to two groups: the VPA-induced autism model group and the AVP group. The rats in the AVP group were treated with intranasal AVP at postnatal day (PND) 21 and for 3 weeks. The VPA-induced autism model group was given the same dose of normal saline in the same way. Behavioral responses were evaluated in the open field and three-chambered social test apparatus; the expression levels of AVP in serum were detected by enzyme-linked immunosorbent assay kit, and the gene expression levels on the amygdala were measured by RNA-seq at PND42.

**Results:**

Intranasal administration of AVP can significantly improve the social interaction disorder and elevate the levels of AVP in serum. Transcriptome sequencing results showed that 518 differently expressed genes (DEGs) were identified in the VPA-induced autism model group compared with the control in this study. Gene Ontology biological process enrichment analysis of DEGs showed that the VPA-induced autism model group had significant nervous system developmental impairments compared with the normal group, particularly in gliogenesis, glial cell differentiation, and oligodendrocyte differentiation. Gene Set Enrichment Analysis (GSEA) enrichment analysis also showed that biological process of oligodendrocyte differentiation, axoneme assembly, and axon ensheathment were inhibited in the VPA-induced autism model group. Pathway enrichment analysis of DEGs between the control and VPA-induced autism model group showed that the PI3K/AKT and Wnt pathways were significantly dysregulated in the VPA-induced autism model group. Few DEGs were found when compared with the transcriptome between the VPA-induced autism model group and the AVP treatment group. GSEA enrichment analysis showed deficits in oligodendrocyte development and function were significantly improved after AVP treatment; the pathways were mainly enriched in the NOTCH, mitogen-activated protein kinase, and focal adhesion signaling pathways, but not in the PI3K/AKT and Wnt pathways. The expression patterns analysis also showed the same results.

**Conclusion:**

AVP can significantly improve the social interaction disorder of VPA-induced autism model, and AVP may target behavioral symptoms in autism by modulating the vasopressin pathways, rather than primary disease mechanisms.

## Introduction

Autism spectrum disorder (ASD) is a complex neurodevelopmental disorder primarily characterized by deficits in social interaction and communication as well as repetitive and stereotypic behavior. The current prevalence rate of ASD is 14.7 per 1,000 children in the United States (Christensen et al., 2016) and 7 per 1,000 in China (Zhou et al., 2020). It is more prevalent in males than in females, with reported ratios ranging from 2:1 to 5:1 (Lord et al., 2020). Although many researches have been carried out, the precise molecular mechanisms of ASD are still not fully elucidated, and no medications are currently approved for ameliorating ASD’s core social behavior deficits.

Arginine vasopressin (AVP) is a neuropeptide hormone synthesized and secreted by separate neuronal populations in the paraventricular nucleus (PVN) of the hypothalamic (Benarroch, 2013; Sparapani et al., 2021). It has been known for several decades that AVP plays a critical role in promoting the formation of complex mammalian social behavior such as social cognition, social recognition, social exploration, and preference by acting on AVP receptors which mainly located in hippocampus, amygdala, striatum, and other “social brains.” In recent years, a growing number of scientific reports have shown that AVP is a crucial factor in neurodevelopmental disorders, including the ASD. Oztan et al. (2018, 2020) (Parker et al., 2018) find that children with ASD have lower cerebrospinal fluid (CSF) AVP concentrations compared with control children and that patients with ASD with the lowest CSF AVP concentrations have the most severe symptoms. Zhang H. F. et al. (2016) find that the plasma AVP levels were associated with repetitive behavior, and ASD children with higher plasma AVP levels tended to have lower levels of repetitive. In addition, lots of researches have shown that correction of vasopressin deficit may ameliorate social deficits of autism (Borie et al., 2021). Parker et al. (2019) presented the safety and efficacy of 4 weeks’ intranasal AVP administration to improve social abilities in children with ASD using a double-blind, randomized, placebo-controlled trial design. Wu et al. (2021) studies have shown that infant rats exposed to VPA showed obviously impaired communication and repetitive behaviors with reduced number of AVP-immunoreactive cells in PVN and content of AVP in CSF. The postnatal subcutaneous injection of AVP can alleviate social preference deficits and stereotyped behaviors, accompanied with the increase in AVP concentrations in the CSF. Clearly, AVP signaling pathway may be a promising therapeutic target for autism. Today, the AVP has already entered phase II clinical trials (NCT01962870, NCT03204786) for the treatment of autism. Although intranasally administered AVP will observably achieve behavioral effects, the precise central mechanisms remain elusive.

To explore the potential for AVP as a treatment for autism and to further explore their mechanisms of action, we compared the amygdala transcriptome changes before and after AVP treatment in VPA-induced autism rat model.

## Materials and Methods

### Materials

#### Chemicals

Valproic acid sodium salt (P4543-10G) was purchased from Sigma-Aldrich Co. (St. Louis, MO, United States). Argipressin (AVP, HY-P0049) was purchased from MedChemExpress. RAT AVP enzyme-linked immunosorbent assay (ELISA) KIT (55R-1951) was purchased from Fitzgerald (United States).

#### Animals

Male and female Wistar rats weighing 270–290 g were obtained from the Department of Experimental Animal Center of Guizhou Medical University. Animals were housed individually with water and chow freely available under a regulated environment (23°C ± 2°C; 50% ± 10% humidity) with a 12/12-h light–dark cycle. All experiments were approved by the Guizhou Medical University Animal Care and Use Committee.

### Methods

#### Animal Model

**FIGURE 1:**
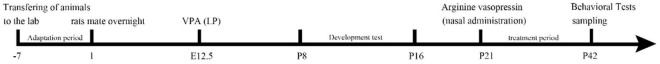
Schematic representation of the experimental procedure.

As previously described (Schneider and Przewlocki, 2005; Dai et al., 2018), female and male rats were allowed to mate overnight, and the morning when spermatozoa were found was designated as embryonic day 0.5 (E0.5). The pregnant rats were randomly distributed into two groups: VPA group (*n* = 10) and saline group (*n* = 5). On E12.5, rats in the VPA group were intraperitoneally injected with sodium valproate (dose of 600 mg/kg, 250 mg/mL dissolved in physiological saline); the saline groups received the same volume of normal saline at the same time. The day of birth of the offspring was marked as postnatal day 1 (PND1). After weaning at PND21, offspring of the same sex were housed separately, with four to five per cage. To assess the treatment effects of AVP, the offspring of VPA group were randomly divided into two groups: VPA-induced autism model groups (*n* = 10) and AVP treatment group (*n* = 10). AVP treatment group received a daily intranasal of AVP (dose of 400 μg/kg, 2.5 mg/mL dissolved in NS) from PND21 to PND42. The offspring of saline groups were marked as control group. The control groups and the VPA-induced autism model group were given the same amount of saline. All experiments were carried out on male offspring. The experimental procedure is shown in Figure 1.

#### Development and Behavioral Tests

##### Open Eyes Test

To examine the maturation process in the pups, we monitored eye opening status from PND12–16 once daily and scored as follows: 0 = both eyes closed, 1 = one eye open, and 2 = both eyes open.

##### Swimming Performance

The swimming test measures motor development and integration of coordinated series of reflex responses (Schneider and Przewlocki, 2005). It was conducted on PND8, PND10, PND12, PND14, and PND16. Each animal was put at the center of an container filled with water (28–29°C) and was observed for 5–10 s. Swimming performance was scored as follows: 0 = head and nose below the surface; 1 = nose below the surface; 2 = nose and top of head at or above the surface, but ears still below the surface; 3 = the same as in 2 except that water line was at mid-ear level; and 4 = the same as in 3 except that water line was at the bottom of ears.

##### Open Field Test

To test for generalized anxiety, the open field test was used at PND42. The tested rat was placed in the center of a squared open field box (80 cm L × 80 cm W × 40 cm H) and allowed to explore freely for 5 min. Locomotion trajectory of the rat was automatically detected and was recorded, and the percentage of the time spent in the central zone in 5 min was calculated.

##### Three-Chamber Test

The three-chamber test was performed at PND42 as previously described (Rein et al., 2020). A polyvinyl chloride box was divided in three compartments (each chamber is 40 cm L × 40 cm W × 40 cm H), and both side compartments contained an empty perforated cup. First, the tested mouse was allowed to explore freely the whole setting, with all doors open for 10 min (phase 1). After this habituation period, the mouse was restricted in the central compartment, whereas an unfamiliar rat of the same sex (stranger 1) was placed under one of the cups (sides alternated between each rat). The tested mouse was then allowed to explore the whole apparatus for 10 min (phase 2). After that, it was restricted to the central compartment, whereas another unfamiliar rat of the same sex (stranger 2) was placed under the other cup. The tested mouse could then again freely explore the whole apparatus for 10 min (phase 3). In all three phases, time spent in each compartment was manually recorded, and the social preference indexes (Rein et al., 2020) were calculated as follows: social preference indexes = (TS − TNS)/(TS + TNS); TS, social stimulus, the interaction time with strange 1 in phase 2 and the interaction time with strange 2 in phase 3; TNS, non-social stimulus, the interaction time in empty in phase 2 and the interaction time with strange 1 in phase 3. To minimize the impact from residual rat odors, the entire apparatus was thoroughly cleaned with 70% ethanol at the beginning of each trial.

##### Self-Grooming Test

The procedure of self-grooming was acquired as described previously, to assess the repetitive behavior at PND42 (Mahmood et al., 2020). In brief, a standard cage of the rat was used to measure repetitive behavior in the self-grooming test. The dimensions of cages were 25 cm wide, 45 cm long, and 20 cm high. After a 5-min habituation in the cage, the cumulative grooming time spent for all body parts of each mouse was calculated by using a stopwatch for 5 min.

### The Expression Levels of Arginine Vasopressin in Serum

Arginine vasopressin content in the serum was assayed using a commercially available ELISA kit (Fitzgerald, 55R-1951).

### RNA-seq

#### Sampling and RNA Isolation

Rats were decapitated, and the amygdala was quickly dissected and frozen in liquid nitrogen for 2 h. The frozen amygdalae were stored at −80°C until used. Total RNA was extracted and purified using TRIzol reagent (Invitrogen, Carlsbad, CA, United States) following the manufacturer’s procedure. The RNA concentration and purity of each sample were quantified using NanoDrop ND-1000 (NanoDrop, Wilmington, DE, United States). RNA quality was measured by Bioanalyzer 2100 (Agilent, Santa Clara, CA, United States). All RNA samples included in the expression analysis had an A260/A280 absorbance ratio greater than 1.8 and RNA integrity number > 7.0.

#### RNA-seq

An RNA-seq library of each sample was prepared, and 2 × 150-bp paired-end sequencing (PE150) was performed on an Illumina Novaseq*™* 6000 (LC-Bio Technology Co., Ltd., Hangzhou, China) following the vendor’s recommended protocol.

#### Sequence Analysis

Raw data files in FASTQ format were generated from the Illumina Novaseq*™* 6000. The reads after quality control and preprocessing were aligned to the reference genome (rattus_norvegicus6.0, v101) using the HISAT2 (Kim et al., 2019) and gene expression quantified using HTSEQ (Anders et al., 2015). The differentially expressed genes (DEGs) were selected with fold changes (FCs) > 1.3 and with statistical significance [false discovery rate (FDR) < 0.05] by DESeq2 (Love et al., 2014); the Gene Ontology (GO) enrichment and Kyoto Encyclopedia of Genes and Genomes (KEGG) enrichment of the DEGs were analyzed using clusterProfiler (Yu et al., 2012). Gene Set Enrichment Analysis (GSEA) was performed to identify the sets of related genes that might be systematically altered in each group by GSEA V4.1.0 (Mootha et al., 2003; Subramanian et al., 2005). The biological process (c5.go.bp.v7.4) and KEGG term (c2.cp.kegg.v7.4) were annotated by Molecular Signatures Database v7.4 (Liberzon et al., 2011, 2015). The significantly enriched gene sets were selected with |NES|>1 (Normalized enrichment score) and with NOM *p* < 0.05 (Nominal *p*-value). Soft clustering was performed using Mfuzz (Kumar and Futschik, 2007) to mine the expression patterns of genes in three groups. In the clustering analysis, the optimal cluster number was calculated using default parameters. Meanwhile, the min score (membership) threshold was set to 0.6. Each cluster was subsequent functional annotation analysis using the online tool g:Profiler^1^ (Raudvere et al., 2019).

#### Quantitative Reverse Transcription–Polymerase Chain Reaction Confirmation

Total RNA from amygdala was extracted utilizing TRIzol reagent (Invitrogen) and reverse transcribed to cDNA using the Thermo Scientific Revert Aid First Strand cDNA Synthesis Kit (Thermo Fisher Scientific Inc., Waltham, MA, United States). Relative expression level of target mRNA was normalized to GAPDH, and relative expression ratio of a target gene was calculated using the mRNA by the 2^–ΔΔCT^ method (Livak and Schmittgen, 2001). The primer sequences used are listed in Table 1.

**TABLE 1:** Primer sequences.

Gene	Primer	Sequence (5′–3′)	Polymerase chain reaction products
Rat GAPDH	Forward	ACAGCAACAGGGTGGTGGAC	253 bp
	Reverse	TTTGAGGGTGCAGCGAACTT	
Rat Olig2	Forward	TGAAGAGACTGGTGAGCGAG	165 bp
	Reverse	GAGGGAGGATGGGGTGATG	
Rat SOX10	Forward	GCAGACGATGACAAGTTCCC	189 bp
	Reverse	CTGGTCGGCTAACTTTCTGC	
Rat MBP	Forward	ATGTGTTTGGGGAGGCAGAT	233 bp
	Reverse	TTGGATGGTCTGAAGCTCGT	

### Statistical Analysis

All data are represented as the mean ± SD. Statistical analysis of multiple-group comparisons was performed by one-way analysis of variance, and two-group comparisons were analyzed by the two-tailed Student *t* test using STATA 14.0 (StataCorp, College Station, TX, United States). Statistical significance was set at *p* < 0.05.

## Results

### Development and Behavioral Tests

#### Eye Opening

**FIGURE 2:**
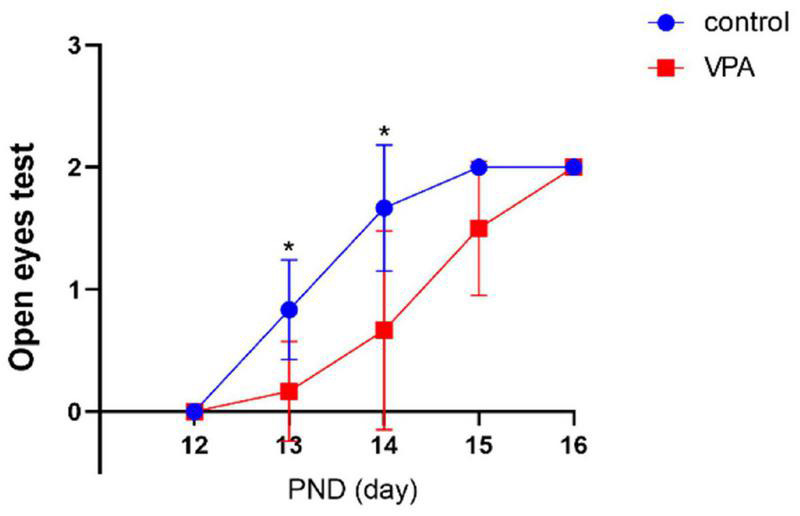
Eye opening test in each group (*n* = 6 in each group). The eye opening score was significantly delayed in the VPA group on PNDs 13 and 14. Compared with the control group: *p < 0.05.

The eye opening scores between the two groups were significantly different on PND13 (*p* < 0.05) and PND14 (*p* < 0.05) with significantly lower eye opening score in the VPA group. On PND12, PND15, and PND16, the eye opening score was similar, and no statistically significant difference was seen between the two groups (Figure 2). The results indicate that VPA may significantly delay the maturation of rats.

#### Swimming Performance

**FIGURE 3:**
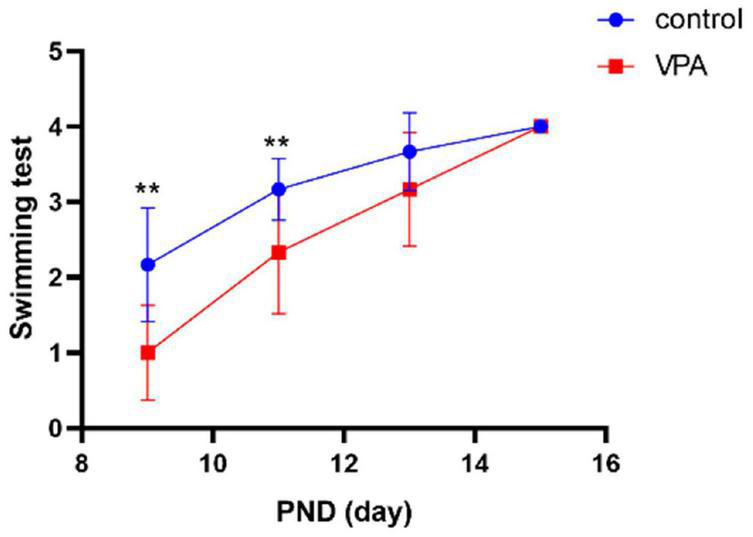
Swimming test in each group (*n* = 6 in each group). The swimming score was significantly delayed in the VPA group on PND9 and PND11. Compared with the control group: **p < 0.01.

Compared with the control group, the ontogeny of swimming behavior was significantly delayed in VPA rats on PND9 (*p* < 0.01) and PND11 (*p* < 0.01), and no significant differences were found on PND13 and PND15 (Figure 3). The results indicate that VPA may significantly delay motor development and attenuate integration of coordinated series of reflexes.

#### Open Field Test

**FIGURE 4:**
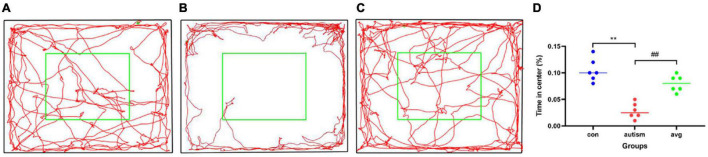
Open field test (*n* = 6 in each group). **(A)** locomotion trajectory of control group, **(B)** locomotion trajectory of VPA-induced autism model group, **(C)** locomotion trajectory of AVP group, **(D)** compare the time spend in center in each group. Compared with the control group: **p < 0.01; compared with the VPA-induced autism model group: ##*p* < 0.01.

When compared with the control group, the anxiety-like behavior was significantly enhanced (it spent more time staying in the edge and corner and less time in the central zone than did the control group) in the VPA-induced autism model group (*p* < 0.01), and the anxiety-like behavior was significantly decreased after the treatment with AVP (*p* < 0.01; Figure 4).

#### Three-Chamber Test

**FIGURE 5:**
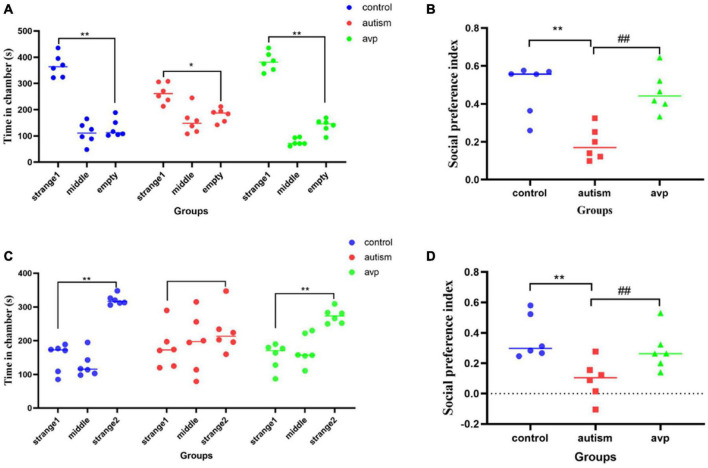
The results of three-chamber test in each group (*n* = 6 in each group). **(A)** The results of social preference test in each group. Comparison of the time spent in the side with stranger 1 vs. the side with empty: *p < 0.05, **p < 0.01. **(B)** Social preference index in social preference test. Compared with the control group: **p < 0.01; compared with the autism group: ##*p* < 0.01. **(C)** The results of social novelty test in each group. Comparison of the time spent in the side with stranger 1 vs. the side with stranger 2: *p < 0.05, **p < 0.01. **(D)** Social preference index in social novelty test. Compared with the control group: **p < 0.01; compared with the autism group: ##*p* < 0.01.

In social preference test (phase2), each group rats spent more time in the side with stranger 1 than in the empty cage (*p* < 0.01). The time in the side with stranger 1 were significantly lower in the VPA-induced autism model group than control (*p* < 0.01) and AVP group (*p* < 0.01; Figure 5A). The social preference index in social preference test was significantly decreased in the VPA-induced autism model group compared with the control group (*p* < 0.01) and AVP group (*p* < 0.01; Figure 5B). In the social novelty test (phase 3), the control and AVP groups spent more time in the side with stranger 2 than in the side with stranger 1 (*p* < 0.01). The VPA-induced autism rats seemed to lose interest in social novelty with conspecifics, spending a comparable time exploring the cage containing stranger 1 rat and stranger 2 (*p* > 0.01; Figure 5C). Furthermore, the social preference index in social novelty test was significantly decreased in the VPA-induced autism model group compared with the control group and AVP group (*p* < 0.01; Figure 5D). The results suggest that the social interaction was impaired in the VPA-induced autism model group, and the impairment was significantly improved following AVP treatment.

#### Self-Grooming Test

**FIGURE 6:**
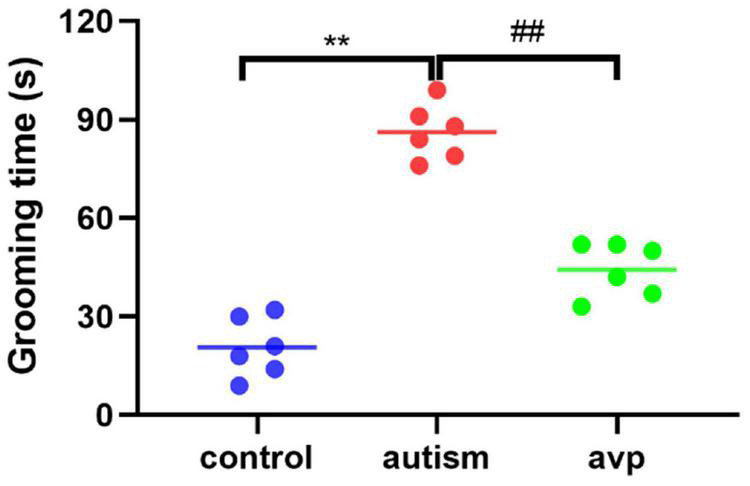
Repetitive/stereotypic behavior test in rats (*n* = 6 in each group). Compared with the control group: **p < 0.01; compared with the VPA-induced autism model group: ##*p* < 0.01.

To test for repetitive/stereotypic behavior, the self-grooming test was used. When compared with the control group, the cumulative self-grooming time was significantly prolonged in the VPA-induced autism model group (*p* < 0.01), and the cumulative time was significantly shorter in the AVP treatment group (*p* < 0.01) when compared with the VPA-induced autism rats (Figure 6).

### The Expression Levels of Arginine Vasopressin in Serum

**FIGURE 7:**
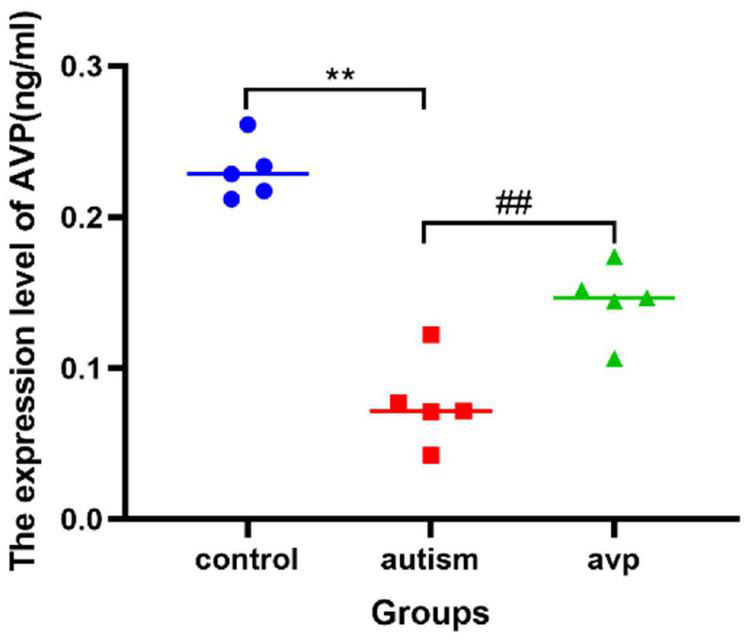
The expression levels of serum AVP in each group (*n* = 5 in each group). Compared with the control group: **p < 0.01; compared with the VPA-induced autism model group: ##*p* < 0.01.

Compared with the control group, the AVP level in the serum significantly decreased in the VPA-induced autism model group (*p* < 0.01). After AVP treatment, the AVP levels in the serum were significantly increased (*p* < 0.01; Figure 7).

### RNA-seq

#### Quality Control and Read Mapping

The number of total reads, clean reads, the sequencing error rate, and the percentage of Q20, Q30, and the GC content of all samples met the quality control requirements of sequencing. More than 95% of the clean reads were mapped to the rat genome, and more than 75% of them were mapped to a single genome location.

#### Differentially Expressed Analysis

**FIGURE 8:**
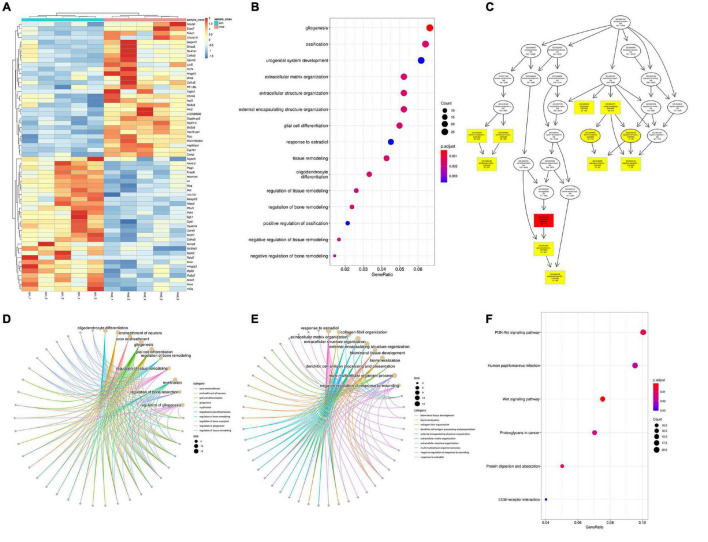
Differentially expressed analysis in autism group compared with the control group (*n* = 5 in each group). **(A)** Hierarchical clustering heatmap of the top 50 DEGs in the VPA-exposed group compared with the control group. **(B)** GO biology process enrichment analysis of the DEGs. The *x* axis indicates the enrichment ratio, and the *y* axis indicates the GO terms. The size of the circle represents the gene number, and the color of the circle indicates the value (adjusted *p* value). **(C)** Directed acyclic graph of GO biology process in DEGs, the depth of the color represents the degree of enrichment. **(D,E)** GO biology process enrichment analysis of down-regulated and up-regulated genes. The size of the circle represents the gene number, and the color of the line indicates clustering of biological processes **(F)** KEGG enrichment analysis of DEGs. The *x* axis indicates the enrichment ratio, and the *y* axis indicates the KEGG terms. The size of the circle represents the gene number, and the color of the circle indicates the value (adjusted *p* value).

There are 518 genes (216 up and 302 down) whose expression was significantly different with an adjusted FDR *p* < 0.05 and FC > 1.3 (only 20 upregulated and 7 downregulated genes were found in FC > 2) in the VPA-induced autism model group compared with the control (the heatmap of the top 50 DEGs are shown in Figure 8A). We performed GO biology process (BP) and KEGG pathway functional enrichment analyses to explore the potential biological functions of these differential expression genes. The results of GO BP were significantly enriched in nervous system development, tissue development and remodeling, and extracellular structure organization (Figures 8B,C). Clearly, the VPA-induced autism model group had significant developmental impairments compared with the control group, particularly in the nervous system [gliogenesis, glial cell differentiation, and oligodendrocyte (OL) differentiation]. Further analysis showed that neurodevelopmental disorders and tissue developmental disorders were associated with down-regulated genes, whereas extracellular matrix remodeling was associated with up-regulated genes (Figures 8D,E). The KEGG pathway enrichment analysis showed that these differential expression genes were significantly enriched in six pathways, such as PI3K-Akt signaling pathway, Wnt signaling pathway, protein digestion and absorption, and so on (Figure 8F). We also analyzed the intersection between our differential expression gene list and the list of 1,010 genes that have evidence of genetic association with ASD from the SFARI human gene list^2^. Of 518 differential expression genes, 27 were found in the SFARI database, which are mainly involved in nervous system development, synapse maturation, synapse organization, protein localization to synapse, glial cell migration, and so on.

There are nine genes (seven up and two down) whose expression was significantly different with an adjusted FDR *p* < 0.05 and FC > 1.3 (no significantly different genes were found in FC > 2) in the VPA-induced autism model group compared with the AVP group. The number of DEGs between the VPA-induced autism model and the AVP group was limited and too few for confident GO and KEGG annotation.

#### Gene Set Enrichment Analysis

Traditional KEGG analysis strategies usually focus on a handful of gene that exhibit differences between two states of interest. Although useful, they are easily affected by the filter threshold (logFC and FDR) and lead to miss some genes with moderate differential expression but important biological significance. To overcome many genes with moderate but meaningful expression changes are discarded by the strict cutoff value, which leads to a reduction in statistical power, we conducted a GSEA (Subramanian et al., 2005). From a biological perspective, GSEA methods are promising because functionally related genes often display a coordinated expression to accomplish their roles in the cell. In the VPA-induced autism model group compared with the control, the results of GSEA GO BP enrichment analysis showed that 239 gene sets are significantly upregulated in the VPA-induced autism model group (NOM *p* < 0.05, |NES|>1). The top 5 terms were significantly enriched in mitochondrial electron transport NADH to ubiquinone, ATP synthesis coupled electron transport, positive regulation of telomerase RNA localization to Cajal body, regulation of cellular amino acid metabolic process, and oxidative phosphorylation. Two hundred eighty-seven gene sets are significantly downregulated in the VPA-induced autism model group (NOM *p* < 0.05, |NES|>1). The top 5 GO terms were significantly enriched in OL differentiation, axoneme assembly, axon ensheathment in central nervous system (CNS), negative regulation of glial cell differentiation, and microtubule bundle formation. The results of GSEA KEGG enrichment analysis showed that 18 gene sets are significantly upregulated in the VPA-induced autism model group (NOM *p* < 0.05, |NES|>1). The top 5 terms were significantly enriched in proteasome, Parkinson’s disease, oxidative phosphorylation, Alzheimer’s disease, and Huntington disease. Four gene sets (base excision repair, ether lipid metabolism, notch signaling pathway, and peroxisome proliferator–activated receptor [PPAR] signaling pathway) were significantly downregulated in the VPA-induced autism model group (NOM *p* < 0.05, |NES|>1).

In the VPA-induced autism model group compared with the AVP group, the results of GSEA GO BP enrichment analysis showed that 96 gene sets are significantly upregulated in the VPA-induced autism model group (NOM *p* < 0.05, |NES|>1). The top 5 GO terms were significantly enriched in neuropeptide signaling pathway, γ-aminobutyric acid (GABA) signaling pathway, synaptic transmission GABAergic, chromatin remodeling at centromere, and establishment of mitochondrion localization. Six hundred sixty-six gene sets are significantly downregulated in the VPA-induced autism model group (NOM *p* < 0.05, |NES| > 1). The top 5 GO terms were significantly enriched in positive regulation of response to cytokine stimulus, positive regulation of interleukin 4 production, cellular extravasation, cell differentiation involved in metanephros development, and regulation of lymphocyte homeostasis. The results of GSEA KEGG enrichment analysis showed that nine gene sets are significantly upregulated in the VPA-induced autism model group (NOM *p* < 0.05, |NES|>1). The top 5 terms were significantly enriched in neuroactive ligand–receptor interaction, O glycan biosynthesis, aminoacyl tRNA biosynthesis, proteasome, and citrate cycle (tricarboxylic acid cycle). Twenty-seven gene sets are significantly downregulated in the VPA-induced autism model group (NOM *p* < 0.05, |NES|>1). The top 5 pathways were significantly enriched in complement and coagulation cascades, *Leishmania* infection, cytokine–cytokine receptor interaction, prostate cancer, and basal cell carcinoma.

**FIGURE 9:**
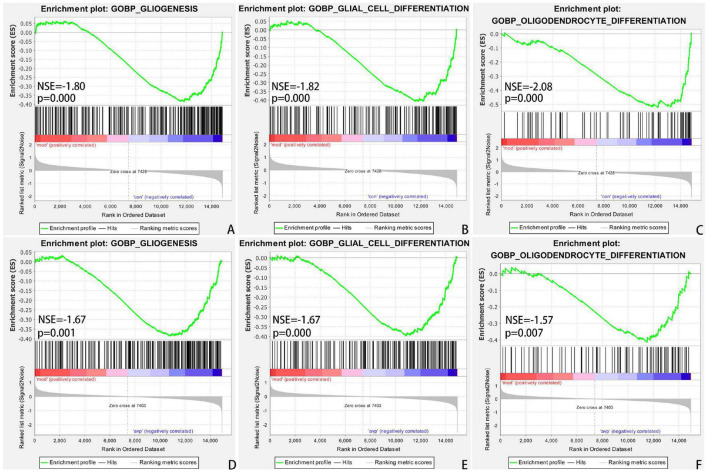
Enrichment of related biological process by GSEA. **(A)** Gliogenesis in control (con) vs. autism (mod), **(B)** glial cell differentiation in control (con) vs. autism (mod), **(C)** oligodendrocyte differentiation in control (con) vs. autism (mod), **(D)** GLIOGENESIS in AVP vs. autism (mod), **(E)** glial cell differentiation in AVP vs. autism (mod), and **(F)** oligodendrocyte differentiation in AVP vs. autism (mod). The significantly enriched gene set was selected with |NES|>1 and with NOM *p* < 0.05.

**FIGURE 10:**
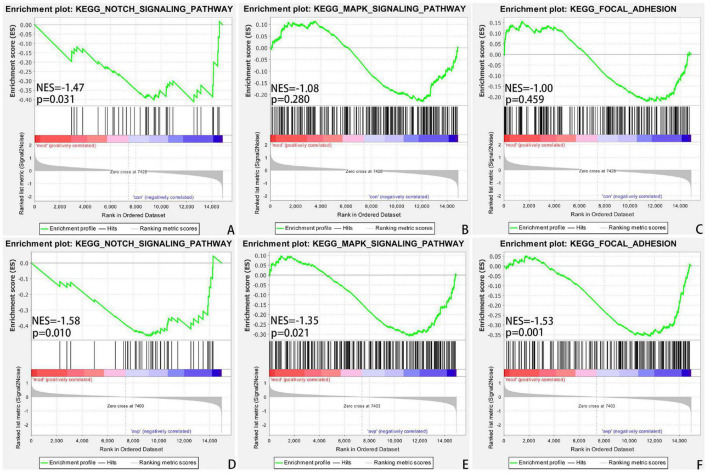
Enrichment of related signaling pathway by GSEA. **(A)** Notch signaling pathway in control (con) vs. autism (mod). **(B)** MAPK signaling pathway in control (con) vs. autism (mod). **(C)** Focal adhesion signaling pathway in control (con) vs. autism (mod). **(D)** Notch signaling pathway in AVP vs. autism (mod). **(E)** MAPK signaling pathway in AVP vs. autism (mod). **(F)** Focal adhesion signaling pathway in AVP vs. autism (mod). The significantly enriched gene set was selected with |NES|>1 and with NOM *p* < 0.05.

The intersection of the gene expression variation between down in the VPA-induced autism model group (con vs. mod) and up in AVP (mod vs. AVP) was analyzed by Evenn^3^. The results showed that 102 BPs were remarkably downregulated in the VPA-induced autism model group and upregulated in AVP treatment, such as OL differentiation, glial cell differentiation, gliogenesis, and so on (Figures 9A–F). Furthermore, some neurodevelopment-related BPs such as glial cell proliferation, glial cell migration, neural tube development, and so on were not changed in the VPA-induced autism model group but remarkably upregulated after AVP treatment. Only notch signaling pathway was found remarkably downregulated in the VPA-induced autism model group and upregulated in AVP treatment. Some neurodevelopment-related pathways, such as mitogen-activated protein kinase (MAPK) signaling pathway, focal adhesion, and so on, were not changed in the VPA-induced autism model group but remarkably upregulated after AVP treatment (Figures 10A–F).

#### Dynamic Expression Patterns of Genes

**FIGURE 11:**
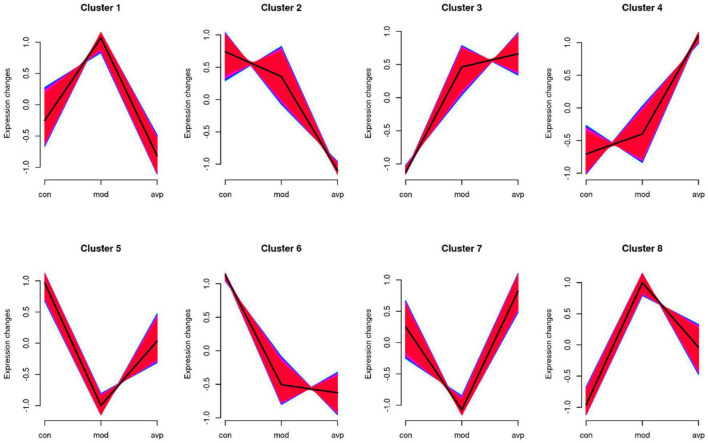
The series of diagrams illustrates the patterns of dynamic changes of gene in each group. Clusters 1 and 8 presented a trend of increasing in the VPA-induced autism model group and decreasing in the AVP group. Clusters 5 and 7 presented a trend of decreasing in the VPA-induced autism model group and increasing in the AVP group. Clusters 2 and 6 presented a trend of gradually declining in the VPA-induced autism model group and AVP group, and clusters 3 and 4 presented a trend of continuous increase in the VPA-induced autism model group and AVP group.

The expression trends of genes at each group were analyzed using Mfuzz clustering analysis, and eight clusters with different change trends were screened out (Figure 11). Genes in clusters 1 and 8 presented a trend of increasing in the VPA-induced autism model group and decreasing in the AVP group. Genes in clusters 5 and 7 presented a trend of decreasing in the VPA-induced autism model group and increasing in the AVP group. Genes in clusters 2 and 6 presented a trend of gradually declining in the VPA-induced autism model group and AVP group. Genes in clusters 3 and 4 presented a trend of continuous increase in the VPA-induced autism model group and AVP group.

We further analyzed the gene biology function in clusters 5 and 7. A large number of development-related biological processes are enriched in cluster 5, such as neurodevelopment, neurogenesis, gliogenesis, glial cell differentiation, and migration, and these genes all point together to notch signal pathway. In cluster 7, lots of genes were enriched in the cellular developmental process, cell differentiation, cell adhesion, cell migration, and so on, and these genes were mainly involved in the focal adhesion pathway and MAPK pathway. The results of expression trend analysis were basically consistent with those of analysis of GSEA.

#### The mRNA Levels of Oligodendrocyte and Myelin Development-Related Genes

**FIGURE 12:**
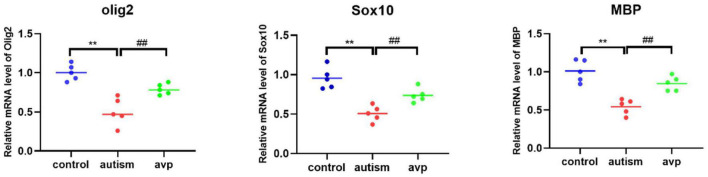
The mRNA levels of oligodendrocyte and myelin development-related genes (*n* = 5 in each group). Compared with the control group: **p < 0.01; compared with the VPA-induced autism model group: ##*p* < 0.01.

Compared with the control group, the mRNA levels of OL and myelin development-related genes, such as Sox10 (a transcription factor that directs neural stem cells toward the glial lineage), Olig2 (a transcription factors necessary for OL development), and MBP (a structural component of myelin, expressed exclusively by myelinating glia), were significantly decreased in the VPA-induced autism model group (*p* < 0.05), and the mRNA levels were improved after AVP treatment (*p* < 0.05) (Figure 12).

## Discussion

Our study showed that rats exposed to VPA at E12.5 had significantly delayed growth development and impaired social behaviors, and the social behaviors were improved significantly by AVP treatment. The results of this study are consistent with previous reports (Wu et al., 2021).

As a hub in the “social brain,” the amygdala, which was located within the anterior medial portion of the brain’s temporal lobe, maintains diverse connections with the neocortex, basal ganglia, hippocampus, thalamus, and other brain cortical areas *via* a network of white matter tracts, responsible for information processing that subserve emotional learning, social cognition, and social interaction (Baron-Cohen et al., 2000; Zalla and Sperduti, 2013; Ferrara et al., 2021; Raam and Hong, 2021; Xu et al., 2021). A large number of studies have demonstrated that amygdala structural and/or functional abnormalities are associated with social malfunctioning in ASD (Shen et al., 2016; Avino et al., 2018; Herrero et al., 2020; Xu et al., 2020; Seguin et al., 2021; Vitor-Vieira et al., 2021). So, we speculated that the prosocial behavior of AVP may be related to the amygdala. In order to further explore its mechanism of action, we focused on transcriptome analysis in the amygdala after AVP treatment.

### Deficits in Oligodendrocyte Development and Function Potentially Represent a Common Molecular Pathway Disrupted in Autism Spectrum Disorder

Five hundred eighteen DEGs were identified in the VPA-induced autism model group compared with the control in this study. GO biological process enrichment analysis of DEGs showed that the VPA-induced autism model group had significant nervous system developmental impairments compared with the normal group, particularly in gliogenesis, glial cell differentiation, and OL differentiation. GSEA enrichment analysis also showed that the biological process of OL differentiation, axoneme assembly, and axon ensheathment were inhibited in the VPA-induced autism model group. Further analysis showed that neurodevelopmental disorders were associated with the transcription inhibition of OL development and myelination-related genes. The results were consistent with the research of Zhang X. F. et al. (2020) who found the transcript levels of myelin-related genes were reduced in poly(I:C)-exposed mouse offspring in the MIA model. Obviously, deficits in OL development and function may be one of the core mechanisms of autism. This may be related to VPA directly inhibits histone deacetylase (Kataoka et al., 2013), causing transient hyperacetylation in the brain, inhibiting development-related genes transcription (Kataoka et al., 2013; Taleb et al., 2021), causes a delay in OL differentiation and hypomyelination. In addition, VPA exposure may impairs repair of DNA damage (Servadio et al., 2018), modifies cholesterol/isoprenoid metabolism, and reduces the number of OLs leading to lower myelin and cholesterol levels (Cartocci et al., 2018).

OLs are glial cells in the CNS, responsible for myelin sheath formation, which allows fast signal transmission, provides metabolic support to axons (Cherchi et al., 2021), and contributes to the optimal information processing in complex neural networks to maintaining the functional connectome of the brain. Deficits in OL development and function will lead to demyelination and axonal dysregulation, and disruption in neuron–glia interactions promotes autistic-like features (Bronzuoli et al., 2018; Duncan et al., 2021). Ample evidence has revealed that OL development disorders are closely related to autism. Graciarena et al. (2018) found that reduction in the proliferation of oligodendroglial cells and low levels of myelin basic protein has also been implicated in ASD pathogenesis. The analyses of DEGs highlighted OL dysregulation, which we confirmed in two additional mouse models of syndromic ASD (Phan et al., 2020). Zhang X. F. et al. (2020) found oligodendroglia-related gene mRNA, which are critically involved in myelination and OL progenitor differentiation, is significantly decreased in amygdala in maternal immune activation ASD model. Astrocytes and OLs may contribute to neurochemical imbalances described in the autistic brain by disrupting neurotransmission or modifying axonal conduction (Voineagu et al., 2011; Gupta et al., 2014).

### PI3K/AKT and Wnt Pathway May Be the Core Mechanism of Autism

Pathway enrichment analysis of DEGs between the control group and VPA-induced autism model group showed that the PI3K/AKT and Wnt pathways were significantly dysregulated in the VPA-induced autism model group.

#### PI3K/AKT Signaling Pathway Represents an Essential Signaling Mechanism for Mammalian Enzyme-Related Receptors

PI3K/AKT signaling pathway represents an essential signaling mechanism for mammalian enzyme-related receptors in transducing signals or biological processes such as cell development, differentiation, cell survival, protein synthesis, and metabolism (Sharma and Mehan, 2021). Up to now, there is still not a unified conclusion on the expression changes of PI3K/Akt pathway in autism. Nicolini et al. (2015) find that PI3K–AKT–mTOR signaling pathways were downregulated in idiopathic autism fusiform gyrus and in neocortex of valproic acid–induced rat model. Luo et al. (2020) find that the PI3K/Akt pathway was inactivated in BTBR mice; overexpressed moesin significantly restored the activity of PI3K/Akt pathway and improved the autistic-like behaviors. Tylee et al. (2017) found that PI3K–AKT–mTOR and RAS–MAPK signaling cascades were diminished, and ribosomal translation and natural killer cell–related activity increased in ASD blood transcriptome. Conversely, Zhang J. et al. (2016) find that p-PI3K and p-AKT significantly increased in the hippocampus in autism rat model, and inhibition of mTOR by NVP-BEZ235 significantly reduced the activity of PI3K/Akt and improved social interaction in VPA-induced ASD. Zhang W. et al. (2020) find that small GTPase gene RAB39b mutation promotes PI3K–AKT–mTOR activity and alters cortical neurogenesis, leading to macrocephaly and autistic-like behaviors. The inconsistent results may be due to the sample size, different brain regions, different developmental time, and different research methods.

### Wnt/β-Catenin Signaling Pathway Plays a Central Role in Neurodevelopment

Wnt/β-catenin signaling pathway plays a central role in neurodevelopment, and perturbation Wnt signaling may trigger the advent of disorders related to the structures and functions of the CNS (Inestrosa and Varela-Nallar, 2015; Kumar et al., 2019). Zhang et al. (2012) (Qin et al., 2016; Kuo and Liu, 2018) find that prenatal administration of VPA causes the upregulation of the Wnt/β-catenin signaling pathway, which facilitates susceptibility to autism. Sulindac treatment ameliorates autism-like behavioral phenotypes probably at least in part *via* the downregulation of the canonical Wnt/b-catenin (Zhang et al., 2015). Vallee et al. (2019) found Wnt/β-catenin pathway was upregulated in autism, and PPARγ agonists may play potential treatment for ASD by inhibiting the canonical Wnt/β-catenin pathway. Emery (2010) found the Wnt/β-catenin pathway plays an important role in OL development; mutant mice with elevated Wnt/β-catenin signaling in the OL lineage display blocked differentiation and hypomyelination. It is obvious that the PI3K/AKT and Wnt pathways may be the core mechanism of autism; targeting these pathways can be beneficial for the improvement of autism (Sharma and Mehan, 2021).

### Arginine Vasopressin Improves the Autism-Like Behavior Through Non-core Pathways

Although AVP treatment significantly improved social interaction disorders in VPA-induced autism model rats, the mechanism remains unclear. Few DEGs were found when compared with the transcriptome between the VPA-induced autism model group and AVP treatment group. GSEA enrichment analysis showed that deficits in OL development and function were significantly improved after AVP treatment, the pathways were mainly enriched in the NOTCH, MAPK, focal adhesion signaling pathways, but not in the PI3K/AKT and Wnt pathway. The expression patterns analysis also showed the same results. Thus, we speculate that AVP can compensate for the dysregulation of PI3K/AKT and Wnt pathways by regulating the NOTCH, MAPK, and focal adhesion signaling pathways, thus promoting OL development and myelin formation and improving autism-like behavior. A large number of studies have confirmed that activation of NOTCH (Kim et al., 2020; Li et al., 2021), MAPK (Ishii et al., 2016; Lorenzati et al., 2021), and Focal adhesion (Forrest et al., 2009; Lafrenaye and Fuss, 2010) signaling pathway can regulate the proliferation, differentiation, and migration of OL and myelin formation. In addition, crosstalk between these pathways and PI3K/AKT and Wnt pathway has been reported in many neurological or non-neurological disorders (Bai et al., 2019; Ishii et al., 2019, 2021; Worthmuller and Ruegg, 2020; Acar et al., 2021).

## Conclusion

Collectively, these results demonstrate that AVP can significantly improve the social interaction disorder of VPA-induced autism model, and AVP may target behavioral symptoms in autism by modulating the vasopressin pathways, rather than primary disease mechanisms.

## Data Availability Statement

The datasets presented in this study can be found in online repositories. The names of the repository/repositories and accession number(s) can be found in the article/supplementary material.

## Ethics Statement

The animal study was reviewed and approved by Guizhou Medical University.

## Author Contributions

BZ, LT, and MW: conceptualization, writing, supervision, project administration, and funding acquisition. XZ, JP, and YZ: animal model. XZ, XY, and YL: behavioral tests. BZ and YC: RNA-seq data analysis. All authors read and approved the final manuscript.

## Conflict of Interest

The authors declare that the research was conducted in the absence of any commercial or financial relationships that could be construed as a potential conflict of interest.

## Publisher’s Note

All claims expressed in this article are solely those of the authors and do not necessarily represent those of their affiliated organizations, or those of the publisher, the editors and the reviewers. Any product that may be evaluated in this article, or claim that may be made by its manufacturer, is not guaranteed or endorsed by the publisher.

## Footnotes

1
https://biit.cs.ut.ee/gprofiler/gost

2
https://gene.sfari.org/database/human-gene/

3
http://www.ehbio.com/test/venn/#/
